# ASSESSING ERGONOMIC RISKS: REBA ANALYSIS OF FOOD DELIVERY RIDERS IN EASTERN PENINSULAR MALAYSIA

**DOI:** 10.13075/ijomeh.1896.02558

**Published:** 2025

**Authors:** Aziah Daud, Ijlal Syamim Mohd Basri, Elyas Ahmad, Suhaily Mohd Hairon, Rusli Nordin, Nor Azali Azmir, Mohd Azlis Sani Md Jalil

**Affiliations:** 1 Universiti Sains Malaysia, Department of Community Medicine, School of Medical Sciences, Kelantan, Malaysia; 2 MAHSA University, Faculty of Medicine, Bioscience and Nursing, Selangor, Malaysia; 3 Universiti Tun Hussein Onn, Faculty of Mechanical and Manufacturing Engineering, Johor, Malaysia

**Keywords:** REBA, WMSDs, ergonomic risk, food delivery riders, motorcycle ergonomics, Eastern Peninsular Malaysia

## Abstract

**Objectives:**

Rapid urbanization has intensified the demand for human labor, including in sectors like food delivery, where workers are prone to work-related musculoskeletal disorders (WMSDs). This study aimed to assess the ergonomic risks among food delivery riders in Eastern Peninsular Malaysia using the Rapid Entire Body Assessment (REBA) method.

**Material and Methods:**

A cross-sectional study was conducted among 191 food delivery riders in September 2021 – March 2022. The REBA method, a paper-and-pen observational tool, was utilized to evaluate the postural risks associated with WMSDs. Data on demographics and working conditions were collected through interviews and observations. Descriptive statistics were used to summarize REBA scores, with mean scores compared between motorcycle types using t-tests.

**Results:**

The mean final REBA score was 5, indicating a medium risk for developing WMSDs. Scores of 4, 5, and 6 were observed in 36.1%, 31.4%, and 31.9% of riders, respectively. Riders using scooters generally had lower REBA scores than those using sedan motorcycles.

**Conclusions:**

The study highlighted a medium risk of WMSDs among food delivery riders. Ergonomic interventions, particularly in motorcycle design, are necessary to mitigate these risks and improve occupational safety and health.

## Highlights

- A medium ergonomic risk among food delivery riders based on the Rapid Entire Body Assessment (REBA) analysis was identified.
- Scooter users showed lower REBA scores compared to sedan motorcycle users.
- High-powered motorcycle users exhibited a high ergonomic risk (REBA score: 9).
- Different motorcycle types significantly influence riders' ergonomic risk levels.
- Targeted ergonomic interventions are recommended to reduce risk of work-related musculoskeletal disorders.

## INTRODUCTION

Rapid urbanization has increased the demand for human labor, particularly in small to medium-scale industries. Despite the introduction of machinery, manual labor remains prevalent, contributing to the high incidence of work-related musculoskeletal disorders (WMSDs) globally. Work-related musculoskeletal disorders have been reported as the most common work-related illness among occupational diseases in various countries, including Japan, Nordic countries, and the USA [1–3]. While WMSDs are not solely attributable to working conditions, numerous studies have identified several occupational factors that predispose workers to these disorders. The main contributing factors include prolonged static positions, unergonomic working postures, and extended exposure to vibration [4,5]. Recent research has further emphasized the impact of psychosocial factors and organizational aspects on the development of WMSDs [6].

Food delivery riders represent a frequently overlooked workforce that is highly susceptible to WMSDs. In Malaysia, food delivery services have emerged as a significant employment option, particularly for those who lost their jobs during the COVID-19 pandemic. The sector was designated as an essential service during the Movement Control Order (MCO), a nationwide lockdown implemented to curb the spread of COVID-19, leading to a substantial increase in food delivery riders [7]. This rapid growth has raised concerns about these workers' occupational health and safety, with recent studies highlighting the ergonomic challenges they face [8]. However, there were still limited data regarding WMSDs among this group. Nevertheless, a study conducted by Hafzi et al. [9] revealed that occupational motorcyclists (OMCs) had a significantly higher prevalence of musculoskeletal issues compared to non-occupational motorcyclists (NMCs), at 82.3% and 62.8%, respectively. This disparity could be attributed to prolonged exposure to static positions due to extended riding times. Additionally, the study found that OMCs had higher mean posture scores, potentially leading to a greater prevalence of WMSDs, particularly lower back pain (LBP).

Given these concerns, there is a pressing need for ergonomic assessments to quantify the magnitude of the problem and develop preventive measures. With the advancement of technologies and research, the methods of evaluating WMSDs have become more accurate from time to time. Current assessment methods can be categorized into 4 groups: paper and pen observational methods, computer-assisted analysis, self-reported assessment tools, and direct instrumental methods [10]. Among these, paper and pen observational methods remain widely used in ergonomic risk assessment due to their cost-effectiveness, ease of implementation, and time efficiency. The potential impact of this research in this field is significant and can lead to substantial improvements in occupational health and safety.

The Rapid Entire Body Assessment (REBA) is a popular paper-and-pen observational method used to assess workers' postural risk of developing WMSDs. It was introduced by Hignett and McAtamney [11] to assess the postural risk of developing WMSDs among workers. In addition, this systematic method is highly reliable and sensitive to WMSDs in various occupational fields, with a reliability rate of 62–85% [12]. The advantages of REBA methods are that they are cost-effective, easy to conduct, and the assessment covers almost every part of the body position. In addition, the REBA method was widely used in many sectors, such as manufacturing, agriculture, forestry, fishing and others. Applying ergonomic risk assessment using the REBA method helps the industries sustain longer due to positive prevention indicators revealed through this method [13].

## MATERIAL AND METHODS

### Study design, study site and setting

This cross-sectional study involved 191 food delivery riders in Eastern Peninsular Malaysia. The sample size was calculated using 2 proportion formula, with α of 0.05 and 80% power of study. Participants were randomly selected using the snowball method, with Captain Rider – a representative leader among food delivery riders – as the initial seed. All participants were chosen based on specific study criteria. The study's objectives were thoroughly explained to the riders, and written consent was obtained before participation. Ethical approval was granted from Human Research Ethics Committee of Universiti Sains Malaysia (JEPeM code: USM/JEPeM/21030230).

### Study period

The study was conducted in September 2021 – March 2022.

### Study population

The target population consisted of all registered food delivery riders in Eastern Peninsular Malaysia.

### Study criteria

Participants included all registered food delivery riders without congenital musculoskeletal disorders (MSDs).

### Data collection

**Figure 1.**
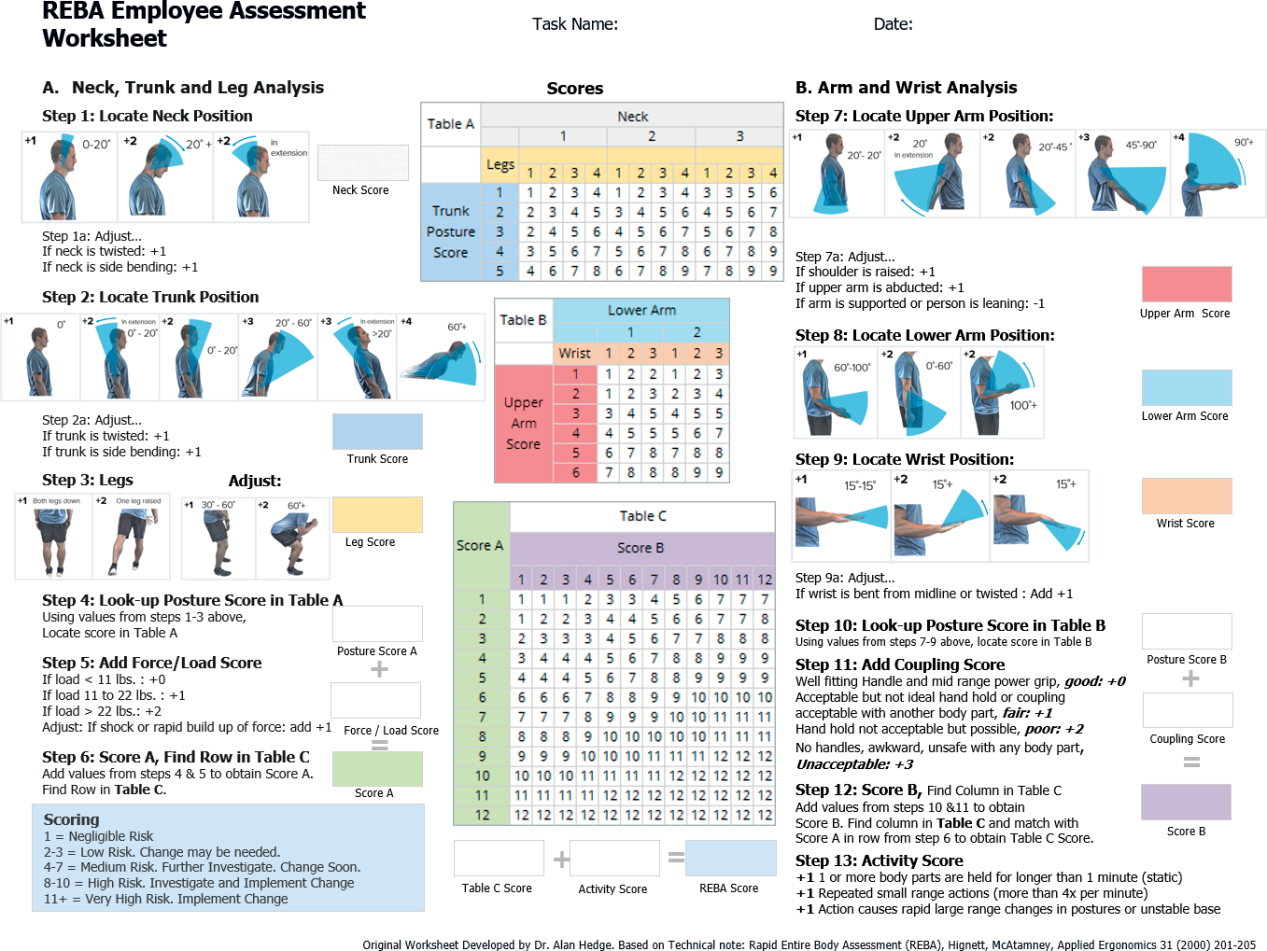
Rapid Entire Body Assessment (REBA) employee assessment worksheet

A total of 191 food delivery riders participated in the study. The REBA assessment sheet was employed to evaluate the postural risk of various body parts, including the neck, trunk, legs, upper arms, lower arms, and wrists. The data collection process involved the following steps:

1) video recording – each rider was videotaped while riding their motorcycle; the videos were then paused to capture specific working postures;
2) scoring using REBA – the captured postures were assessed using the REBA sheet, which includes 3 sections: score A, score B, and the final REBA score:
  – score A: This section evaluates the neck, trunk, and legs. The neck position was scored as 1 or 2, with adjustments for twisting or side bending. The trunk position was scored 1–4 based on the angle from the body centre, with twisting or bending adjustments. The leg posture was scored as 1 or 2, with adjustments if knee flexion angles were 30–60° or >60°. The final score A was determined using table A, incorporating the neck, trunk, and leg scores along with the load score;
  – score B: This section assesses the arms and wrists. The upper arm position was scored 1–4 based on the degree of elevation, with adjustments for raised shoulders, abducted upper arms, and supported or leaning positions. The lower arm posture was scored as 1 or 2, and the wrist posture was scored as 1 or 2, with adjustments for bending or twisting. The final score B was determined using the upper arm, lower arm, and wrist scores, along with the coupling score;
  – final REBA score: The final score was calculated using table C, which combines scores A and B with the activity score. All steps were summarized in Figure 1.

These steps were repeated for all 191 food delivery riders.

## RESULTS

### Demographic data

This study assessed 191 food delivery riders in Eastern Peninsular Malaysia, comprising 177 males (92.7%) and 14 females (7.3%), with an average age of 27.6 years. The riders' work experience varied, with 92 riders (48.2%) having 6–12 months of experience and 99 riders (51.8%) having >12 months. On average, riders completed 18.9 trips/day, worked 10.2 h/day and 6.1 days/week. The majority of riders used sedan motorcycles (83.8%), followed by scooters (15.7%), and only one rider used a highpowered motorcycle (0.5%). The characteristics of the respondents' results are presented in Table 1.

**Table 1.** Characteristics of food delivery riders, Eastern Peninsular Malaysia, September 2021 – March 2022

Variable	Participants (N = 191)		M±SD
	n	%	
Gender			
male	177	92.7	
female	14	7.3	
Age [years]			27.6±5.76
Working experience			
6–12 months	92	48.2	
>12 months	99	51.8	
Trips [n/day]		18.9±6.16	
Working time [h/day]			10.2±2.33
Working time [days/week]			6.1±1.03
Type of motorcycle			
sedan	160	83.8	
scooter	30	15.7	
high-powered	1	0.5	

### REBA score

**Figure 2.**
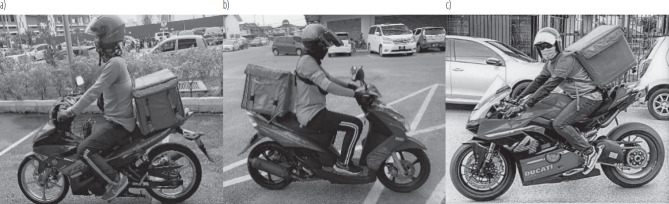
Working posture in motorcycle types: a) sedan, b) scooter, and c) high-powered

The rider's individual final REBA score was measured using a REBA assessment sheet based on individual posture, as shown in Figure 2.

Photo: Elyas Ahmad

The ergonomic assessment using the REBA method revealed the following distribution of final REBA scores: 69 riders (36.1%) scored 4, 60 riders (31.4%) scored 5, and 61 riders (31.9%) scored 6. One rider (0.5%) had a score of 9 as shown in Table 2. The mean final REBA score was 4.98 (SD = 0.88), indicating a medium risk level for developing MSDs. The level of MSD risk was categorized based on the score as in Table 3.

**Table 2.** Final Rapid Entire Body Assessment (REBA) scores among food delivery riders, Eastern Peninsular Malaysia, September 2021 – March 2022

REBA score	Participants (N = 191)	
	n	%
4	69	36.1
5	60	31.4
6	61	31.9
9	1	0.5

**Table 3.** Level of musculoskeletal disorders (MSD) risk based on final Rapid Entire Body Assessment (REBA) score, Eastern Peninsular Malaysia, September 2021 – March 2022

REBA score	MSD risk
1	negligible, no action required
2–3	low risk, change may be needed
4–7	medium risk, further investigation, change soon
8–10	high risk, investigate and implement change
≥11	very high risk, implement change

When analyzing REBA scores by motorcycle type, it was found that among sedan users (N = 160), 54 riders (38.8%) scored 4, 48 riders (30.0%) scored 5, and 58 riders (36.3%) scored 6. Among scooter users (N = 30), 15 riders (50.0%) scored 4, 12 riders (40.0%) scored 5, and 3 riders (10.0%) scored 6. The single high-powered motorcycle user had a score of 9.

Regarding the final REBA score, 69 riders (36.1%) had a final score of 4, while 60 riders (31.4%) had a final score of 5, and 61 riders (31.9%) had a final score of 6 as shown in Table 2. Only 1 rider had a final score of 9. In addition, the mean final REBA score was calculated and concluded as 5. Upon further investigation, among riders who rode sedan motorcycles, 38.8% of riders had a final REBA score of 4, 30.0% – a score of 5, and 36.3% – a score of 6. Meanwhile, among those who rode scooters, the final REBA score of 4, 5, and 6 were 50.0%, 40.0% and 10.0%, respectively. Finally, the highest score, which was 9, belongs to the rider who rode a high-powered motorcycle, as summarized in Table 4.

**Table 4.** Crosstab motorcycle type and final Rapid Entire Body Assessment (REBA) score, Eastern Peninsular Malaysia, September 2021 – March 2022

Motorcycle type	Participants (N = 191) [n (%)]				
	total	score 4	score 5	score 6	score 9
Sedan	160 (83.8)	54 (38.8)	48 (30.0)	58 (36.3)	0
Scooter	30 (15.7)	15 (50.0)	12 (40.0)	3 (10.0)	0
High-powered	1 (0.5)	0	0	0	1 (100.0)

These findings highlight that the majority of food delivery riders are at medium risk for MSDs, with scooter users generally having lower REBA scores compared to sedan users. The type of motorcycle appears to influence the ergonomic risk levels, suggesting a need for targeted ergonomic interventions to reduce the risk of MSDs among food delivery riders.

## DISCUSSION

Ergonomics among workers has become a crucial aspect of occupational health, aiming to enhance the well-being of workers across various industries. The rapid increase in the number of food delivery riders in Malaysia has raised urgent concerns about their ergonomic risks. This study revealed that different types of motorcycles significantly influence REBA scores due to varying postures, aligning with findings that REBA scoring is highly sensitive to different working postures [14].

This study also revealed that almost all riders had a final REBA score ranging 4–6 and a mean final REBA score of 5. According to Table 3, the level of MSD risk for food delivery riders in Terengganu was medium risk based on their final REBA score. The same result was revealed in a study by Montolalu et al. [15] among online transportation drivers in Indonesia, where the final REBA score was 7. Further investigation and the need to change workstations are necessary to improve the ergonomic posture of the riders. However, modifying the workstation for motorcycle riders presents unique challenges, such as the need to consider the dynamic work environment and the specific tasks involved in food delivery. Recent research by Dabholkar et al. [16] emphasizes the importance of adapting ergonomic assessment tools for specific occupations, particularly those with dynamic work environments like food delivery. This suggests that tailored approaches may be necessary for effectively addressing the ergonomic risks faced by food delivery riders. The study identified an outlier with a final REBA score of 9 for a high-powered motorcycle user, indicating a high risk of MSDs. This finding underscores the variability of REBA scores across different work settings, as documented in various ergonomic studies [17–19]. The major contribution of the high score comes from score A. In depth, the rider's neck was in extension position and the trunk was leaned forward in almost 50° position. In addition, the rider had load bearing >5 kg which came from the helmet, bag and food inside the bag that need to be carried along the journey to deliver the food for customer. In contrast, the load bearing by the scooter and sedan users were <5 kg as they did not have to carry the delivery bag on their back because it can be fitted on the seat of the motorcycle. With the final REBA score of 8 make the high-powered motorcycle user had high risk of developing WMSDs. Furthermore, based on the risk table, there was a need of further investigation and change must be implemented as soon as possible to prevent greater risk and consequences from WMSDs. Thus, there should be an urgent action to quantify the individual WMSDs status among the riders especially on the high-powered motorcycle users as they were highly susceptible to get WMSDs based on the working posture alone.

This study also revealed that scooter users tend to have lower final REBA scores, where only 10% had a score of 6, and the majority had a final REBA score of 4 even though the MSD risk was still the same. Compared to sedan users, the distribution of the scores was relatively homogenous from score 4 to 6. The reason for this is due to the riders' different leg scores. Scooter users tend to have similar leg posture, which explains the pool of final REBA score in score 4. In contrast, different ranges of leg postures demonstrated in sedan users give rise to the homogeneity of the final REBA score in those 3 scores. A study by Karuppiah et al. [20] further supports this, highlighting how different vehicle types can significantly impact ergonomic risks for motorcycle riders. The primary contributing factors to these variations in scores were trunk and neck posture, where scooter users maintained a more upright position, while sedan users demonstrated a wider range of postural adjustments, resulting in greater score variability. The high final REBA score among food delivery riders predisposed the riders to develop WMSDs. This explained why OMCs had a higher prevalence of WMSDs as compared to NMCs because they were exposed to unergonomic posture for longer duration [9]. On top of that, this study also showed that riding a high-powered motorcycle predisposed the riders to a higher risk of getting WMSDs. This indicates that high-powered motorcyclists should be discouraged from being food delivery riders as the risk of developing WMSDs is high.

To reduce the risk of WMSDs among the riders, further investigation must be done to improve the ergonomic posture among the riders. This proactive approach, using the hierarchy of control, emphasizes the need for continuous improvement in the field of occupational health. Technological advancement must fully utilize engineering control to improve motorcycle ergonomics. For example, the tendency to lean forward, which affects the trunk score, can be enhanced by improving the seat height, handlebar height and footrest position [21]. Li et al. [22] suggested that a comprehensive approach combining ergonomic interventions with organizational and individual-level strategies may be most effective in addressing the complex ergonomic challenges faced by food delivery riders.

### Strengths and limitations

Using the REBA method in research offers several strengths, making it a valuable tool for ergonomic studies. Rapid entire body assessment provides a systematic and quantitative approach to assessing postural risks associated with various tasks, enabling researchers to identify specific areas of concern and prioritize interventions effectively. Its versatility allows for the evaluation of a wide range of occupations and activities, ensuring broad applicability. Additionally, REBA's straightforward scoring system facilitates easy interpretation and communication of results, promoting evidence-based decision-making in ergonomic interventions.

This study has several limitations that warrant consideration. This study has several limitations. First, its crosssectional design limits causal inferences between ergonomic risk factors and WMSDs. A larger, representative study across different regions in Malaysia is needed for broader generalizability. While REBA provides an overall risk assessment, it does not differentiate risk levels for individual body parts. Splitting scores could introduce inaccuracies. Future studies using biomechanical analysis and motion capture could offer more detailed insights. Additionally, this study does not analyze the impact of food bag placement and weight on musculoskeletal strain. Future research should explore force distribution and load-bearing assessments. The authors have provided initial insights into weight distribution effects in the discussion. Despite these limitations, this study significantly enhances understanding of ergonomic risks among food delivery riders. It underscores the need for ergonomic interventions, such as seat height, handlebar positioning, and footrest design adjustments. Future research will focus on developing a tailored WMSD prevention module for food delivery riders.

## CONCLUSIONS

In conclusion, this study reveals a pressing concern for the ergonomic well-being of food delivery riders in Eastern Peninsular Malaysia, evidenced by a mean final REBA score of 5, indicating a medium risk of WMSDs. The differential impact of motorcycle types on ergonomic risk is particularly notable: scooter users generally experience lower REBA scores compared to sedan users, likely due to superior postural support offered by scooter designs. Alarmingly, a high-powered motorcycle user recorded a final REBA score of 9, signaling a high risk of WMSDs and underscoring the significant impact of the authors' findings on the urgent need for targeted interventions. It is recommended that collaboration with motorcycle manufacturers be pursued to optimize rider postures and mitigate ergonomic risks, contributing to the overall well-being of food delivery riders.
